# Effects of dietary and exercise treatments on HDL subclasses in lactating women with overweight and obesity: a secondary analysis of a randomised controlled trial

**DOI:** 10.1017/S0007114522000241

**Published:** 2022-12-14

**Authors:** Elisabeth Adolfsen Øhman, Lisa Kirchner, Anna Winkvist, Fredrik Bertz, Kirsten Bjørklund Holven, Stine Marie Ulven, Hilde Kristin Brekke

**Affiliations:** 1 Department of Nutrition, Institute of Basic Medical Sciences, University of Oslo, Oslo, Norway; 2 Department of Internal Medicine and Clinical Nutrition, The Sahlgrenska Academy, University of Gothenburg, Gothenburg, Sweden; 3 Norwegian National Advisory Unit on Familial Hypercholesterolemia, Department of Endocrinology, Morbid Obesity and Preventive Medicine, Oslo University Hospital, Oslo, Norway

**Keywords:** Dietary treatment, Diet, Exercise, Postpartum, Weight loss, HDL, Overweight

## Abstract

Childbearing decreases HDL-cholesterol, potentially contributing to the increased risk of CVD in parous women. Large HDL particles (HDL-P) are associated with lower risk of CVD. In this secondary analysis of a randomised controlled trial, we investigated the effects of 12-week dietary and exercise treatments on HDL-P subclass concentration, size and apoA1 in lactating women with overweight/obesity. At 10–14 weeks postpartum, 68 women with pre-pregnant BMI 25–35 kg/m^2^ were randomised to four groups using 2 × 2 factorial design: (1) dietary treatment for weight loss; (2) exercise treatment; (3) both treatments and (4) no treatment. Lipoprotein subclass profiling by NMR spectroscopy was performed in serum at randomisation and after 3 and 12 months, and the results analysed with two-way ANCOVA. Lipid concentrations decline naturally postpartum. At 3 months (5–6 months postpartum), both diet (*P* = 0·003) and exercise (*P* = 0·008) reduced small HDL-P concentration. Concurrently, exercise limited the decline in very large HDL-P (*P* = 0·002) and the effect was still significant at 12 months (15 months postpartum) (*P* = 0·041). At 12 months, diet limited the decline in very large HDL-P (*P* = 0·005), large HDL-P (*P* = 0·001) and apoA1 (*P* = 0·002) as well as HDL size (*P* = 0·002). The dietary treatment for weight loss and the exercise treatment both showed effects on HDL-P subclasses in lactating women with overweight and obesity possibly associated with lower CVD risk. The dietary treatment had more effects than the exercise treatment at 12 months, likely associated with a 10 % weight loss.

Childbearing is associated with increased risk of CVD^(1–3)^ and a decreased level of HDL-cholesterol that persists for decades^(1,4,5)^. After the first pregnancy, HDL-cholesterol has been shown to decrease and remain lowered^(6)^. Low concentration of HDL-cholesterol is considered an independent predictor of CVD in epidemiological research^(7–10)^. Genetic and randomised clinical trials, the latter most often drug-based, have failed to demonstrate that increasing HDL-cholesterol levels can prevent CVD events^(11–14)^. Recently, evidence suggests that HDL particle (HDL-P) concentration is a better marker than HDL-cholesterol for risk of CVD events in people both with and without a CVD history^(15,16)^. This measure may depict HDL properties better than does HDL-cholesterol as the different sizes of the HDL-P are heterogeneous in biological function, content and intravascular metabolism, for example, do larger subclasses of HDL carry more cholesterol than do smaller subclasses^(17,18)^. New advanced methods have made it possible to measure the concentration, size and lipid content of lipoprotein particles in blood samples from observational and clinical studies^(19)^. The results from these studies may contribute to improved risk prediction of CVD and a better understanding of how alterations in lipid metabolism can cause metabolic diseases^(20)^.

The prevalence of overweight and obesity is still increasing globally, also in women of fertile age. Among women in developed countries, almost 30 % in the age category 20–29 years and > 50 % in the age category 45–49 years were affected by overweight or obesity in 2013^(21)^. Women with high pre-pregnancy weight have an elevated risk of excess postpartum weight retention^(22)^ and postpartum metabolic syndrome^(23)^. BMI and percent body fat increase with increasing parity, and postpartum weight retention increases BMI for each pregnancy in developed countries^(1,24)^. Obesity is strongly correlated with low HDL-cholesterol^(25)^ and explains part of the increased risk of CVD in parous women^(1,5)^. Diet is the evidence-based cornerstone for weight reduction among postpartum women, although exercise is recommended simultaneously for fat-free mass preservation and improvement of cardiorespiratory fitness^(26,27)^. The postpartum period is suggested as a ‘window of opportunity’, since many women are motivated to lose weight and achieve a healthier lifestyle after becoming a parent^(28,29)^. Lactation increases energy requirement and may contribute to weight loss^(30)^. Nevertheless, lifestyle treatment postpartum for women with overweight or obesity is not standard in the Swedish health care system.

In a randomised controlled trial (RCT) in Sweden, a dietary treatment (minus 2100 kJ/d) in lactating women with BMI 25–35 kg/m^2^ resulted in a 9 % weight loss after 3 months and 10 % after 12 months^(31)^. In the same study, an exercise treatment produced neither a significant weight loss nor change in body composition and did not preserve muscle mass. After 12 months, HDL-cholesterol decreased less in the women receiving the dietary treatment compared with the women not receiving the dietary treatment^(32)^. In this secondary analysis, we aimed to explore the observed favourable effect of the dietary treatment on HDL-cholesterol. Our hypothesis was that the dietary treatment also affected the size distribution and composition of the HDL-P, analysed with NMR spectroscopy, as seen in other weight loss studies^(33)^. We also hypothesised that the exercise treatment had no effect on the same variables, as the treatment had no effect on HDL-cholesterol or other blood lipids in our previous analyses^(32)^.

## Methods

### Subjects

Women (*n* 76) with self-reported pre-pregnancy BMI 25–35 kg/m^2^ were recruited between April 2007 and May 2010 from fifteen antenatal clinics in Sweden^(31)^. Inclusion criteria for participation were singleton term delivery, birth weight of infant > 2500 g, non-smoking, intention to breastfeed for a minimum of 6 months, with a maximum of 20 % of infant energy intake from complementary foods and no serious illness in the mother or infant. Women with mild allergies and stable, medicated hypothyroidism were eligible.

### Ethics

All participants gave written informed consent. The RCT and NMR analyses were approved by the Regional Ethical Review Board, Gothenburg, Sweden (483-06), (276-17), (2020–01255), and the trial conducted according to the principles of the Declaration of Helsinki. The trial is registered at www.clinicaltrials.gov as NCT01343238.

### Study design and intervention

Eligible participants among the seventy-six recruited (*n* 68) were examined at baseline (2–3 months postpartum), after 3 months (5–6 months postpartum) and after 12 months (15 months postpartum). Randomisation, using a random number list, was performed in blocks of four within each stratum of either BMI ≥ 28·0 or < 28·0 kg/m^2^. After completing baseline measurements, the women were randomised (using sealed envelopes) to one of the following groups in a 2 × 2 factorial design:

#### 1) Dietary behaviour modiﬁcation treatment

The goal of the dietary treatment was to lose 0·5 kg per week during the 12-week intervention, that is, a weight loss of 6 kg in total and to improve the quality of the diet in line with the Nordic Nutrition Recommendations. This pace of weight reduction is compatible with lactation^(31,34)^. Women in this group received a total of 2·5-h individual dietary treatment with a registered dietitian. The dietary intervention was based on a 4-d food diary and designed to reduce energy intake with 2100 kJ/d. Participants were instructed to follow a stepwise plan to introduce dietary changes and self-monitor their body weight using a digital scale at home. Every 2 weeks, the participants received a cell phone text message asking them to report the latest measured body weight and was followed by feedback on their performance.

#### 2) Physical exercise behaviour modiﬁcation

The goal of the exercise treatment was to implement a 45-min walk at 60–70 % of maximal heart rate (‘pulse zone walk’) four times per week during the 12-week intervention. Women in this group received a total of 2·5-h individual behavioural intervention with a physical therapist. Participants were instructed how to perform walks within the recommended pulse range (as determined during cardiovascular fitness test) using a heart rate monitor (Polar FS2C, Polar Electro Oy). Every 2 weeks, the participants received a cell phone text message asking them to report the number of pulse zone walks performed during the previous week and was followed by feedback on their performance.

#### 3) Dietary and physical exercise behaviour modiﬁcation

The combination group received both the dietary and the exercise treatment as described above.

#### 4) Control group

Women in the control group received usual care, without diet or physical exercise counselling. The women in the control group were offered the dietary treatment after study end. Detailed protocols of the dietary and exercise treatments have been described previously^(35)^.

### Blood samples

Venous blood was drawn after 7–10 h overnight fast at all three visits. Serum was prepared and stored at −70°C at the Department of Internal Medicine and Clinical Nutrition, Sahlgrenska Academy, Gothenburg University. The frozen samples were not thawed during the period of storage.

### Analyses of HDL subclasses

In September 2017, the frozen serum samples were transported on dry ice to Finland for automated high-throughput NMR spectroscopy (Nightingale Health Ltd, Helsinki, Finland), analysing concentration and composition of HDL-P subclasses, apoA1 and apoB and mean size of HDL-P (HDL-D), HDL-cholesterol and HDL-TAG. The HDL-P subclasses were classified in four sizes as follows: very large (XL), large (L), medium (M) and small (S), with average particle diameters of 14·3, 12·1, 10·9 and 8·7 nm, respectively. Detailed description of the NMR spectroscopy methodology is reported elsewhere^(20,36)^.

### Statistical analysis

A sample size calculation was not performed for these secondary analyses. The required sample size of 68 was based on expected weight change for dietary intervention only *v*. control group, which was the primary outcome of the RCT, with power of 80 % and 0·05 two-sided significance level^(31)^.

The 2 × 2 factorial design included two levels (absence or presence) of each of two factors (dietary treatment and exercise treatment), leading to four intervention combinations in total. This design is an efficient use of power as it allows for analysis of main effects of the dietary treatment (diet vs no-diet) and main effects of exercise treatment (exercise vs no-exercise) and potential interaction between treatments. The changes in HDL-related measures from baseline to 3 months and from baseline to 12 months were analysed with two-way ANCOVA, adjusted for baseline values. Significant interaction effects indicate that the effect of one treatment depends on the presence or absence of the other treatment, and vice versa. Such interaction requires analyses of simple main effects, that is, the effect of the treatments both in the presence and in the absence of each other. The significance levels of the simple main effects were manually Bonferroni-adjusted for multiple comparisons. Main effects and simple main effects of the dietary and exercise treatment are reported with corresponding estimated marginal means with standard errors.

Two-way ANCOVA was the preferred statistical method for comparison with previous publications from the same study^(31,32)^. Assumptions for two-way ANCOVA were tested. Statistical signiﬁcance was indicated by *P* < 0·05. IBM SPSS Statistics for Windows version 26.0 was used for all statistical analyses.

## Results

### Subjects

**Fig. 1.**
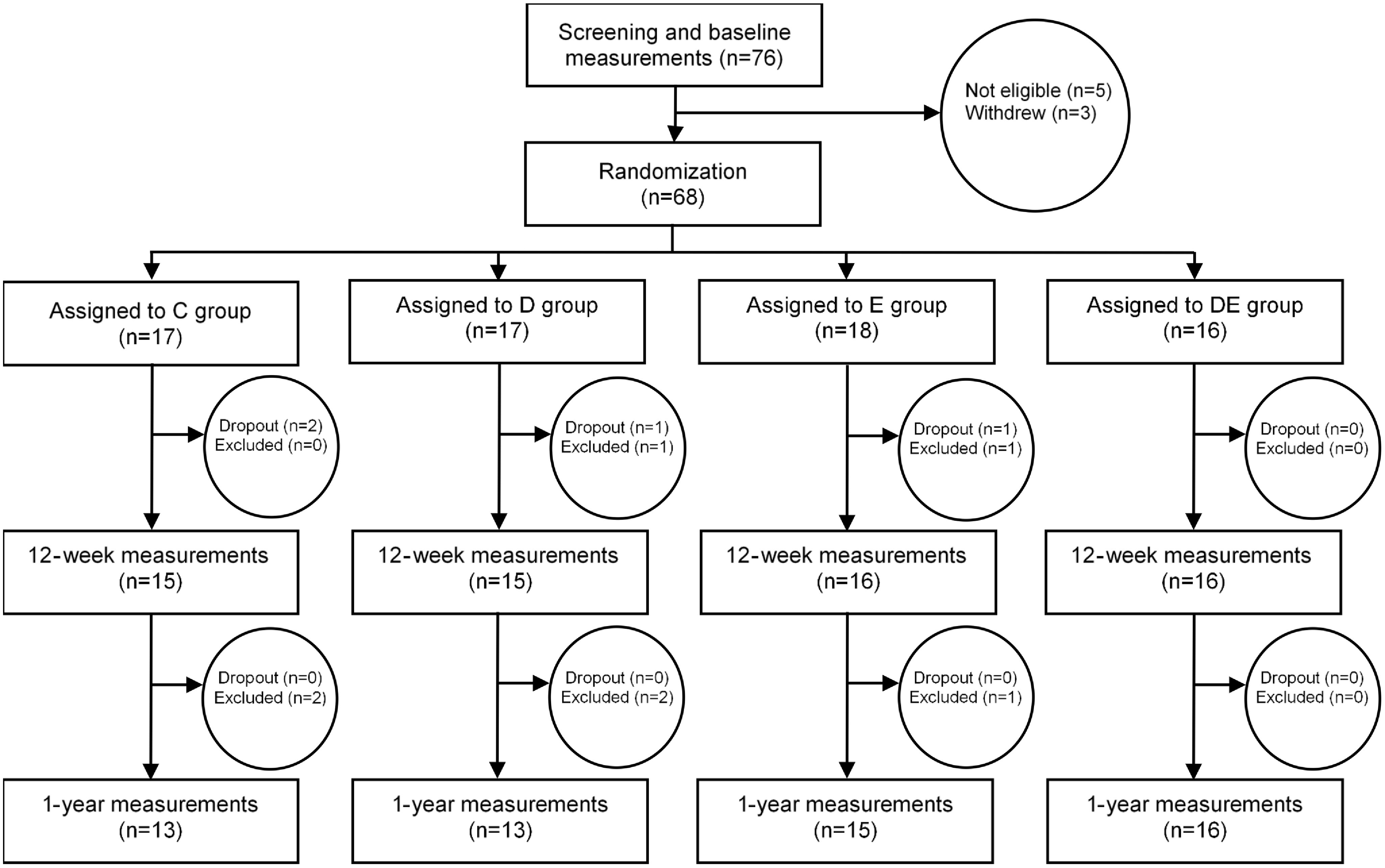
Flow chart of the study participants. C, control; D, dietary treatment; E, exercise treatment; DE, diet and exercise treatments.

Of the sixty-eight randomised women, sixty-two (91 %) completed measurements at 3 months and fifty-seven (84 %) completed the measurements at 12 months (Fig. 1). Reasons for exclusion were new pregnancies for six women, new medication for one woman and four women withdrew for other reasons. The women who received the dietary treatment (diet and diet and exercise groups) reported self-weighing ≥ 2 times per week during the 3-month intervention, and the women receiving the exercise treatment (exercise and diet and exercise groups) reported implementing 86 % of planned walks at recommended 60–70 % of maximal heart rate.

Characteristics and anthropometric and lipid variables at baseline are shown in Table 1 and Table 2. Breast-feeding behaviour and infant growth were similar between the groups both before and after the intervention period^(31)^.

**Table 1.** Characteristics of study participants at baseline 2–3 months postpartum* (Mean values and standard deviations)

	Control (*n* 15)		Diet (*n* 15)		Exercise (*n* 16)		D + E (*n* 16)	
	Mean	sd	Mean	sd	Mean	sd	Mean	sd
Age(years)	32·2	4·6	33·6	4·2	33·2	3·7	33·9	4·2
Parity(*n*)								
1	7		7		9		10	
2	6		8		7		5	
3	2		0		0		1	
Education (*n*)								
Short education at high school	2		0		2		1	
≤ 3 years beyond high school	2		3		3		4	
> 3 years beyond high school	11		12		11		11	
Body weight(kg)	85·5	10·3	85·4	10·0	88·3	11·7	83·8	7·3
BMI (kg/m^2^)	30·2	3·4	30·0	2·6	30·4	3·1	29·9	2·2
Body fat (%)	43·5	5·8	44·6	4·2	42·6	5·1	43·4	5·1

D, diet; E, exercise.

* For normally distributed variables, values are means with standard deviations. For categorical variables, values are frequencies (*n*).

**Table 2.** Particle concentration of HDL subclasses, mean HDL diameter, HDL-cholesterol, HDL-TAG, apoA1 and apoB at baseline 2–3 months postpartum in lactating women with overweight and obesity* (Mean values and standard deviations)

	Control (*n* 15)		Diet (*n* 15)		Exercise (*n* 16)		D + E (*n* 16)	
	Mean	sd	Mean	sd	Mean	sd	Mean	sd
Subclass of HDL particle								
XL-HDL (mol/l (10–06)	0·50	0·12	0·56	0·17	0·45	0·19	0·52	0·18
L-HDL (mol/l (10–06)	1·27	0·25	1·47	0·22	1·18	0·34	1·32	0·44
M-HDL (mol/l (10–06)	2·00	0·28	2·07	0·36	1·96	0·26	2·02	0·29
S-HDL (mol/l (10–06)	4·58	0·41	4·49	0·60	4·59	0·44	4·53	0·41
HDL particle size								
HDL-D (nm)	10·1	0·15	10·2	0·18	10·0	0·20	10·1	0·22
Components								
HDL-cholesterol (mmol/l)	1·50	0·17	1·58	0·19	1·42	0·23	1·51	0·30
HDL-TAG (mmol/l)	0·10	0·03	0·10	0·02	0·10	0·02	0·10	0·03
ApoA1 (g/l)	1·50	0·11	1·54	0·14	1·44	0·15	1·50	0·17
ApoB (g/l)	0·72	0·17	0·70	0·12	0·74	0·17	0·74	0·16

D, diet; E, exercise; XL, very large; L, large; M, medium; S, small; D, diameter; C, cholesterol.

* Values are in means and standard deviation.

### Main and interaction effects of dietary and exercise treatments

Concentrations of most lipids declined between baseline and the two follow-up visits, as expected in the postpartum period.

#### Main effects at 3 months after intervention start

**Fig. 2.**
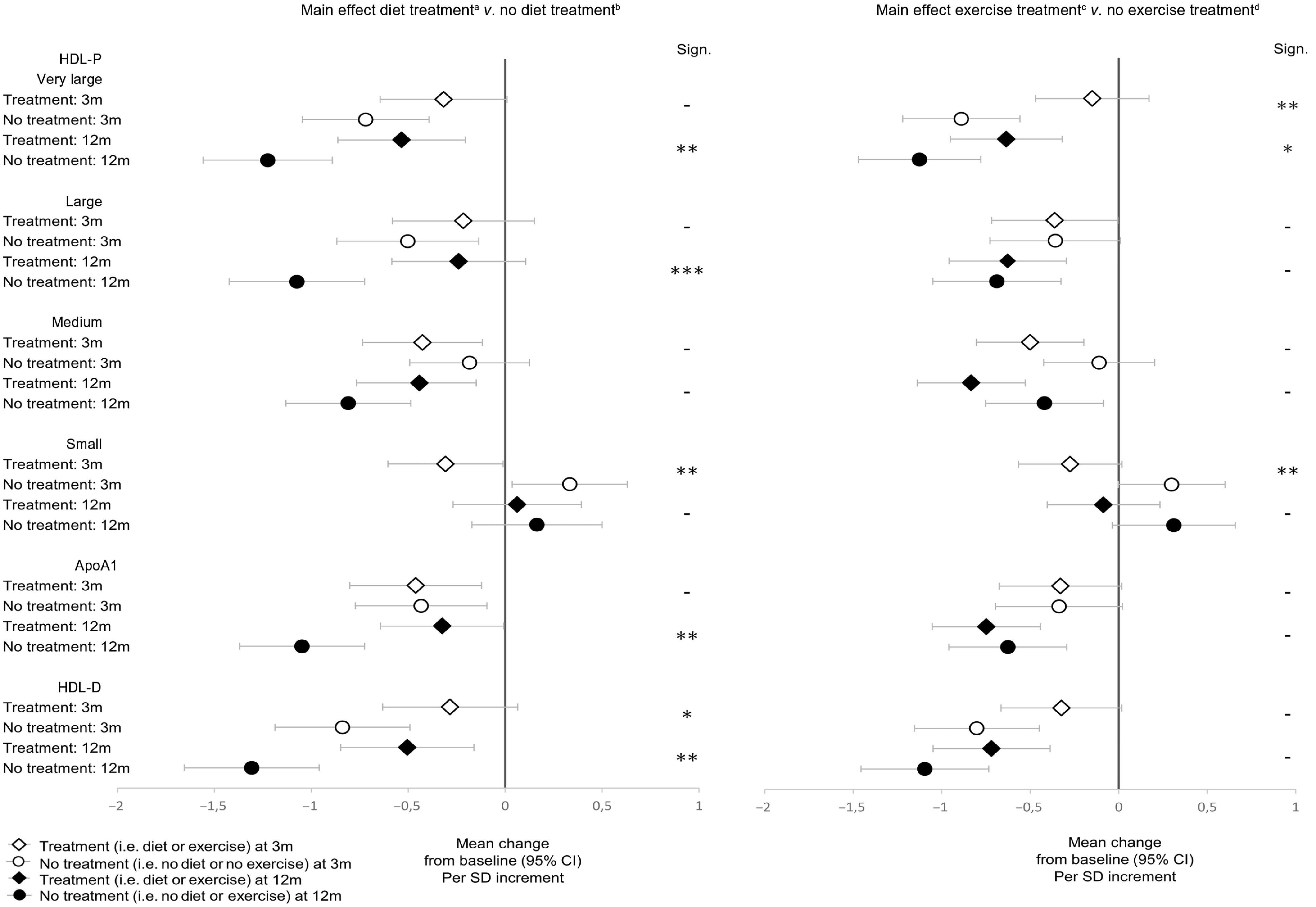
Main effects of diet and exercise treatments *v*. no diet or exercise treatment on concentrations of HDL particles (HDL-P) and apoA1 and mean diameter of HDL (HDL-D) in lactating women with overweight and obesity. The effects are expressed in change at 3 and 12 months after baseline, that is, estimated marginal means from two-way ANCOVA analysis, adjusted for baseline values, per sd increment. Vertical bars represent 95 % CI. ^a^n = 31 at 3 months, *n* 29 at 12 months. ^b^n = 31 at 3 months, *n* 28 at 12 months. ^c^n = 32 at 3 months, *n* 31 at 12 months. ^d^n = 30 at 3 months, *n* 26 at 12 months. *** for *P* ≤ 0·001, ** for *P* ≤ 0·01, * for *P* ≤ 0·05 and – for *P* > 0·05.

Exercise treatment (exercise) limited the decline in the concentration of very large HDL-P (*P* = 0·002) (Table 3, Fig. 2). Both dietary treatment (diet) (*P* = 0·003) and exercise treatment (*P* = 0·008) reduced the concentration of small HDL-P (Table 3, Fig. 2). Diet limited the decline in the mean diameter of HDL (HDL-D) (*P* = 0·030)) (Table 3, Fig. 2). Diet reduced the concentration of apoB (*P* = 0·018) (Table 3).

**Table 3.** Main and interaction effects of dietary and exercise treatments on HDL subclass concentration, HDL particle size and other relevant variables (Mean values and standard errors, P-values <0.05 in bold)

	Estimated marginal means										
	Diet	se	No diet	se	Exercise	se	No exerc	se	*P* main effect		*P* inter-action effect
	*n* 31*		*n* 31*		*n* 32*		*n* 30*		Diet	Exerc.	
Subclass of HDL particle											
XL-HDL (mol/l (10–08)), 3 months	–2·90	1·49	–6·57	1·49	–1·36	1·46	–8·11	1·51	0·090	**0·002**	0·909
XL-HDL (mol/l (10–08)), 12 months	–0·63	0·19	–1·44	0·20	–0·75	0·19	–1·33	0·20	**0·005**	**0·041**	0·702
L-HDL (mol/l (10–07)), 3 months	–0·57	0·48	–1·33	0·48	–0·95	0·47	–0·94	0·49	0·279	0·992	0·208
L-HDL (mol/l (10–07)), 12 months	–0·70	0·50	–3·15	0·51	–1·84	0·48	–2·02	0·53	**0·001**	0·804	0·912
M-HDL (mol/l (10–07)), 3 months	–1·32	0·048	–0·57	0·048	–1·54	0·047	–0·34	0·048	0·271	0·080	**0·037** †
M-HDL (mol/l (10–07)), 12 months	–1·38	0·49	–2·51	0·50	–2·59	0·47	–1·30	0·052	0·110	0·072	0·828
S-HDL (mol/l (10–07)), 3 months	–1·07	0·52	1·16	0·52	–0·95	0·51	1·04	0·52	**0·003**	**0·008**	**0·047** ‡
S-HDL (mol/l (10–07)), 12 months	0·27	0·71	0·70	0·72	–0·37	0·68	1·34	0·75	0·671	0·097	0·842
HDL particle size											
HDL-D (nm), 3 months	–0·031	0·019	–0·091	0·019	–0·035	0·019	–0·087	0·019	**0·030**	0·057	0·522
HDL-D (nm), 12 months	–0·078	0·026	–0·202	0·027	–0·111	0·025	–0·169	0·028	**0·002**	0·131	0·671
Other relevant variables											
HDL-cholesterol (mmol/l), 3 months	–0·054	0·033	–0·071	0·033	–0·062	0·032	–0·063	0·033	0·710	0·976	0·299
HDL-cholesterol (mmol/l), 12 months	–0·071	0·036	–0·235	0·037	–0·160	0·035	–0·146	0·038	**0·003**	0·786	0·715
HDL-TAG (mmol/l), 3 months	–0·013	0·003	–0·006	0·003	–0·014	0·003	–0·005	0·003	0·164	0·051	**0·029** §
HDL-TAG (mmol/l), 12 months	0·002	0·004	0·002	0·004	0·004	0·004	0·001	0·004	0·977	0·662	0·305
ApoA1 (g/l), 3 months	–0·064	0·023	–0·060	0·023	–0·075	0·023	–0·049	0·024	0·908	0·446	0·161
ApoA1 (g/l), 12 months	–0·049	0·024	–0·159	0·024	–0·113	0·023	–0·095	0·025	**0·002**	0·595	0·593
ApoB (g/l), 3 months	–0·107	0·016	–0·053	0·016	–0·089	0·015	–0·071	0·016	**0·018**	0·425	**0·032** ||
ApoB (g/l), 12 months	–0·047	0·018	–0·055	0·018	–0·034	0·017	–0·069	0·019	0·752	0·174	0·488
Serum-TAG (mmol/l), 3 months	–0·138	0·040	–0·009	0·040	–0·109	0·039	–0·037	0·040	**0·027**	0·207	0·122
Serum-TAG (mmol/l), 12 months	0·008	0·054	0·148	0·055	0·084	0·052	0·071	0·057	0·079	0·870	0·634

XL, very large; L, large; M, medium; S, small; D, diameter.

*P*-values for simple main effects significant if < 0·025 because of adjustment for multiple comparisons: Bonferroni.

* At 12 months, the number of participants declined to: diet treatment, *n* 29; no diet treatment, *n* 28; exercise treatment, *n* 31; and no exercise treatment, *n* 26.

† Significant interaction effect in M-HDL at 3 months. Simple main effect of exercise significant within non-diet groups (*P* = 0·007). Estimated marginal mean (EMM) (se) non-exercise, non-diet = 0·75 (0·68). EMM (se) exercise, non-diet = –1·89 (0·66). Simple main effect of diet, not significant after manual Bonferroni adjustment (*P* = 0·028). EMM (se) non-diet, non-exercise = 0·75 (0·68). EMM (se) diet, non-exercise = –1·44 (0·69).

‡ Significant interaction effect in S-HDL at 3 months. Simple main effects of diet significant within non-exercise groups (*P* < 0·001). EMM (se) non-diet, non-exercise = 2·9 (0·74). EMM (se) diet, non-exercise = –0·82 (0·74). Simple main effect of exercise significant within non-diet groups (*P* = 0·001). EMM (se) non-exercise, non-diet = 2·90 (0·74). EMM (se) exercise, non-diet = –0·58 (0·72).

§ Significant interaction effect in HDL-TAG at 3 months. Simple main effects of diet significant within non-exercise groups (*P* = 0·014). EMM (se) non-diet, non-exercise = 0·004 (0·005). EMM (se) diet, non-exercise = –0·014 (0·005). Simple main effect of exercise significant within non-diet groups (*P* = 0·004). EMM (se) non-exercise, non-diet = 0·004 (0·005). EMM (se) exercise, non-diet = –0·016 (0·005).

|| Significant interaction effect in apoB at 3 months. Simple main effects of diet significant within non-exercise groups (*P* = 0·002). EMM (se) non-diet, non-exercise = −0·020 (0·022). EMM (se) diet, non-exercise = –0·122 (0·022). Simple main effect of exercise not significant within non-diet groups (*P* = 0·037). EMM (se) non-exercise, non-diet = –0·020 (0·022). EMM (se) exercise, non-diet = –0·086 (0·022).

No main effects of treatments were observed on concentrations of other HDL-P or apoA at 3 months (Table 3).

#### Main effects at 12 months after intervention start

Both diet (*P* = 0·005) and exercise (*P* = 0·041) limited the decline in the concentrations of very large HDL-P (Table 3, Fig. 2). Diet also limited the decline in the following variables: the concentration of large HDL-P (*P* = 0·001), HDL-D (*P* = 0·002) and apoA1 (*P* = 0·002) (Table 3, Fig. 2).

No other effects of treatments were observed in apoB nor HDL-P concentrations at 12 months (Table 3, Fig. 2).

#### Effects of treatments on lipids in HDL-P at 3 and 12 months after intervention start

Diet limited the decline in the concentration of HDL-cholesterol at 12 months (*P* = 0·003) (Table 3), confirming the findings from the previous publication^(32)^.

**Fig. 3.**
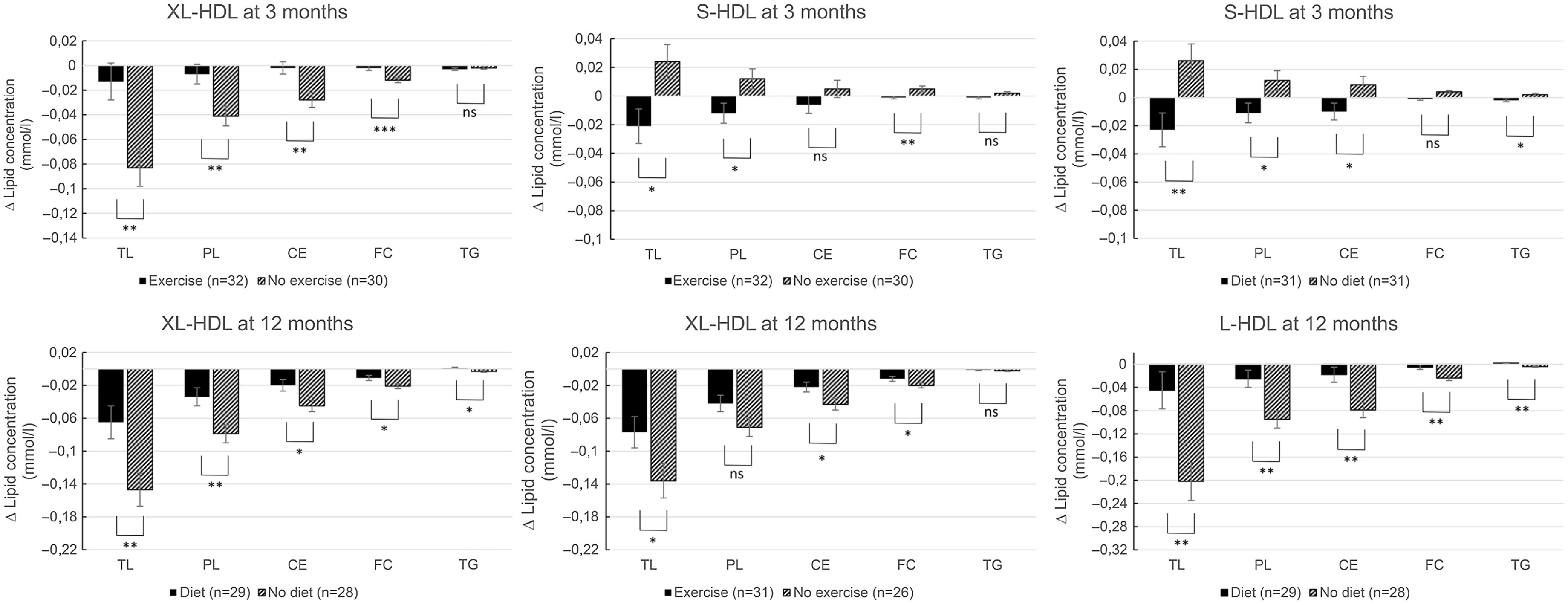
Main effects of diet and exercise treatments on lipid concentrations in the HDL subclasses at time points with significant effects of either of the treatments on the concentration of the HDL subclasses in lactating women with overweight and obesity. The effects are expressed in change of lipid concentration, that is, the estimated marginal means from the to-way ANCOVA analysis. Vertical bars representing standard errors. Abbreviations: TL, total lipids; PL, phospholipids; C, cholesterol, CE, cholesterol esters; FC, free cholesterol. *** for *P* ≤ 0·001, ** for *P* ≤ 0·01, * for *P* ≤ 0·05 and ns for *P* > 0·05.

The changes in the content of total lipids, phospholipids, cholesterol, free cholesterol, cholesterol ester and TAG in the HDL-P subclasses are shown in Fig. 3 and Supplementary Table 1. Dietary treatment (all *P* < 0·040) and to some extent the exercise treatment (all *P* < 0·036) increased or limited the decline in lipids in larger HDL-P and decreased TAG in smaller particle subclasses reflecting the changes seen in HDL-P concentrations.

#### Interaction effects of dietary and exercise treatments

Interaction effects between treatments were observed in the following variables at 3 months: medium HDL-P (*P* = 0·037), small HDL-P (*P* = 0·047), HDL-TAG (*P* = 0·029) and apoB (*P* = 0·032). The interaction effects between treatments did in all cases imply that the treatments were more effective alone than together. Combining treatments rather weakened the effects of each other resulting in the effects of the treatments being significant only in the absence of each other. (Table 3)

#### Percentage change on group level

To illustrate the changes in unadjusted concentrations per group (percent), HDL subclasses, HDL components, HDL-D and apoB from baseline to 3 and 12 months are shown in Table 4. No statistical tests were performed on these unadjusted group level results.

**Table 4.** Percentage change (Δ) per group from baseline to 3 and 12 months follow-up in unadjusted concentrations or size

	Control (*n* 5*)		Diet (*n* 15*)		Exercise (*n* 16*)		D + E (*n* 16*)	
Subclass of HDL particle								
XL-HDL, Δ 3 months (%)†	–20·0	13·5	–13·0	12·5	–6·5	20·9	2·0	19·6
XL-HDL, Δ 12 months (%)‡	–40·8	–43·1, −23·8	–16·1	–32·5, −5·6	–20·9	–43·5, −11·6	–10·0	–16·6, 11·9
L-HDL, Δ 3 months (%)‡	–6·4	–16·0, 2·3	–13·6	–19·7, 6·1	–18·6	–31·4, 15·8	–4·4	–16·2, 16·4
L-HDL, Δ 12 months (%)†	–23·8	21·7	–7·6	18·0	–21·7	25·1	–2·6	20·3
M-HDL, Δ 3 months (%)†	4·9	13·6	–7·3	11·1	–7·9	18·5	–4·9	16·2
M-HDL, Δ 12 months (%)†	–7·2	16·7	–2·8	16·2	–15·3	13·4	–9·4	17·3
S-HDL, Δ 3 months (%)‡	5·0	2·0–9·3	–1·8	–4·9, −3·9	–2·3	–8·1, 3·9	–1·4	–8·3, 0·9
S-HDL, Δ 12 months (%)†	3·8	9·3	4·3	9·2	–0·8	9·1	–0·9	–0·7
HDL particle size								
HDL-D, Δ 3 months (%)†	–1·1	0·8	–0·7	0·7	–0·7	1·3	0·0	1·2
HDL-D, Δ 12 months (%)†	–2·2	1·4	–1·2	1·2	–1·7	1·6	–0·4	1·3
Other relevant variables								
HDL-cholesterol, Δ 3 months (%)†	4·9	16·8	–10·0	20·8	–13·2	26·8	–8·3	21·9
HDL-cholesterol, Δ 12 months (%)†	–15·0	15·5	–4·6	12·4	–13·8	13·4	–5·2	13·0
HDL-TAG, Δ 3 months (%)‡	6·8	–7·1, 17·8	–12·0	–22·4, 3·7	–18·6	–36·1, −1·4	–11·4	–26·4, 13·7
HDL-TAG, Δ 12 months (%)‡	4·0	–13·3, 21·0	1·2	–15·4, 16·9	0·6	–23·4, 13·7	9·7	–8·7, 37·3
ApoA1, Δ 3 months (%)‡	–0·6	–7·43, 5·7	–7·0	–9·4, 2·7	–6·5	–13·7, 1·7	–3·2	–10·3, 0·7
ApoA1, Δ 12 months (%)‡	–12·8	–15·8, −1·1	–4·0	–7·5, 1·4	–8·5	–17·6, 0·7	–4·3	–9·5, 0·5
ApoB, Δ 3 months (%)‡	–1·6	–13·8, 9·3	–14·3	–21·9, −10·7	–15·8	–24·2, 10·0	–10·9	–24·8, −0·3
ApoB, Δ 12 months (%)‡	–10·3	–26·9, 12·2	–8·5	–12·5, 3·1	–9·4	–18·8, 6·4	–7·3	–15·9, 15·6
S-TAG, Δ 3 months (%)‡	7·1	–9·0, 34·8	–10·8	–34·3, 2·5	–18·8	–34·5, 2·5	–19·2	–25·0, 13·9
S-TAG, Δ 12 months (%)‡	18·9	–15·5, 54·3	0·3	–20·1, 40·4	29·6	–3·9, 25·1	10·1	–10·9, 46·2

D, diet; E, exercise; XL, very large; L, large; M, medium; S, small; D, diameter.

* At 12 months, the number of participants declined to: control group, 13; diet group, 13; exercise group, 15; diet and exercise group, 16.

† Normally distributed variables, values are in means and standard deviations.

‡ Non-normally distributed variables, values are in medians and 25–75 percentiles.

## Discussion

The aim of this secondary analysis of the RCT was to explore the HDL-cholesterol-related findings, as the decrease in HDL-cholesterol seen in parous women decades after birth^(1,4,5)^ may contribute to their increased risk of CVD^(1–3)^. The first part of our hypothesis was verified; there was an effect of the dietary treatment on the distribution and composition of the HDL subclasses after intervention. The dietary treatment also affected apoA1 and mean HDL size. However, the other part of our hypothesis was rejected; the exercise treatment did have an effect, although not to the same extent as did the dietary treatment, on the HDL subclasses. Our results extend and support the previously published effects of the dietary treatment on HDL-cholesterol at 12 months post-intervention, decreasing the concentration less in the women receiving dietary treatment compared with the women not receiving dietary treatment^(32)^. In addition, here we report for the first time significant effects of the exercise treatment on blood 0.

Particle concentration of HDL-P subclasses and HDL-cholesterol increase during gestation and decline after delivery, to levels slightly elevated at 3 months postpartum compared with 1–3 years postpartum^(37)^. Baseline concentrations of HDL-P subclasses in our population were similar or slightly elevated compared with non-pregnant, non-lactating women of the same-age group^(38)^. Thus, a decline in the concentration of HDL subclasses was likely ongoing at baseline in our study. To our knowledge, no other studies have reported particle concentration of the HDL subclasses in lactating women.

The exercise treatment significantly limited the decline of very large HDL-P at both time points and reduced the small HDL-P at 3 months after intervention. These findings may be explained by an exercise-induced decrease in liver secretion of very-low-density lipoprotein (VLDL)-TAG and an increase in lipoprotein lipase activity and quantity^(39)^. Consequently, hydrolysis of VLDL-TAG and other TAG-rich lipoproteins increases, inducing a shift from smaller to larger sizes of HDL-P. The purpose of these mechanisms may be to replace intramuscular TAG used during exercise. A meta-analysis of endurance exercise interventions reported an increase in the concentration of the larger HDL-P and a decrease in the concentration of the smaller HDL-P^(39)^. Types of training and duration were comparable to the exercise treatment in our study. The results from the meta-analysis were independent of BMI and change in BMI, indicating that weight loss is not mandatory for metabolic effects of exercise to occur. In our study, there were no effects of exercise treatment on weight loss, number of steps a day or total energy expenditure^(31)^. Since compliance was good, women receiving the exercise treatment in our study may have cut back on other daily activities. The explanation for our findings may be that these women performed more intense activity than the women who did not receive exercise treatment. Thus, the exercise treatment in lactating women caused similar HDL-related effects, independent of weight loss, as seen in other populations^(39)^.

At 12 months, dietary treatment limited the decrease in concentrations of the large and very large HDL-P, HDL-cholesterol and apoA1, variables that showed some of the largest percentage differences between groups. HDL-cholesterol declines during active weight loss^(40)^; even so, we found no significant effect on HDL-cholesterol of any of the treatments at 3 months. However, in the postpartum period, most lipids are generally declining^(37)^, which may have masked possible effects. Lactation seems to limit the decline in HDL-cholesterol^(41,42)^. Most of the women (93 %) were lactating, and there were no associations between treatments and breast-feeding categories^(31)^ which minimises the impact of lactation on HDL-cholesterol treatment effects.

The effect of diet-induced weight loss on maintaining HDL-cholesterol at 12 months was anticipated, as concentration of HDL-cholesterol normally increase when the body weight is stabilised after losing weight^(40)^. In line with other studies^(25)^, effects of the dietary treatment were a decline in serum-TAG in our NMR data concurrent with a reduced fasting insulin and waist circumference at 3 month^(32)^. The decreased fat intake observed in the women with dietary treatment^(35)^ and a possible improved insulin sensitivity may have increased HDL size by a faster postprandial plasma-TAG clearance, that is, hydrolysis of TAG in chylomicrons and VLDL, and thus increased transfer of free cholesterol and phospholipids to HDL^(43)^. Less exposure to serum-TAG decreases CETP-induced exchange of cholesterol esters and TAG between HDL and TAG-rich lipoproteins, contributing to the shift towards larger HDL-P. Our results on lipid content of the HDL subclasses may strengthen these mechanistic theories as the lipid content increased in larger subclasses and TAG decreased in smaller subclasses as an effect of dietary treatment. One trial investigating impact of a dietary intervention for weight loss reported a similar pattern as ours; a short-term reduction in the concentration of smaller HDL subfractions after a 10·5 % weight loss pre-bariatric surgery and an increase in large HDL subfractions following 34 % weight loss 1 year post-bariatric surgery^(33)^. An observational study showed that weight change and HDL-P size were inversely associated^(44)^. Thus, both treatment-induced weight loss and weight reduction in a free-living population seem to result in a shift towards larger HDL-P, probably mediated by improved metabolic fitness.

The HDL-P is dynamic and its activity and size change throughout metabolism. Both smaller and larger particles mediate reverse cholesterol transport, although the large particles more so^(45)^, and both exert other anti-atherogenic actions like anti-inflammatory and anti-thrombotic activity and protect LDL from oxidative modifications^(18)^. Concentration of apoA1, larger and medium HDL-P and HDL size have showed negative associations with incident CVD events in observational studies^(46,47)^, while the concentration of small HDL-P has shown positive association with ischemic stroke^(47)^. HDL size has shown negative association with CVD risk also in a high-risk cardiovascular population^(48)^. Nevertheless, accounting for possible correlations between the subclasses of HDL and established risk factors for CVD, associations may be altered or abolished. In a cohort of healthy females, large and medium HDL-P as well as total HDL-P concentration showed negative association with incident CHD both before and after adjusting for metabolic and lipoprotein variables, including the other HDL subclasses^(49)^. Small HDL-P were positively associated before adjustments, but negatively associated after, while very small HDL-P were positively associated in both analyses. Comparing top quartile with bottom quartile in a case–control study, the inverse association between HDL size and coronary artery disease disappeared after adjustments for levels of apoB and serum-TAG, while the inverse association between total particle concentration of HDL and CAD remained^(50)^. These studies illustrate the importance of considering that other lipids, especially TAG and apoB-containing, atherogenic lipoproteins^(51)^, often affect the correlations between HDL subclasses and CVD. We did not adjust for these variables in our statistical analysis, but neither LDL-C, serum-TAG nor apoB were affected by the treatments at 12 months. ApoA1 is strongly correlated with total HDL-P concentration (*r* = 0·74)^(49)^, which may be a more independent CVD predictor than the concentration of HDL subclasses. Hence, the results of the dietary treatment on HDL-P distribution and composition show CVD-protective associations in epidemiology, although various adjustments show diverging results.

In the variables with interaction effects between treatments, that is, the 3-month effect on small HDL-P, medium HDL-P, HDL-TAG and apoB, one treatment had effect only in the absence of the other treatment. In other words, the effects of the treatments combined are less than the sum of the treatments or even less than the individual treatments alone. A reason for this type of interaction may be that it is challenging to perform both treatments at the same time, or that the effect on blood lipids met a saturation point with one treatment.

This study contributes with unique data as effects of lifestyle treatment on HDL-P subclasses have not previously been reported in this population. Other key strengths of this study are the randomised controlled design and the low dropout that was evenly distributed between treatments^(31)^. The reasons for dropout were mainly new pregnancies and not treatment related. This made it possible to analyse the data in completers only, that is, only women with measurements were included and were analysed irrespective of their adherence to the treatments. There were no missing data points for lipids in non-dropouts.

The study also has limitations. The women in all groups were more active at baseline compared with Swedish women in general^(31)^. A more intense exercise regimen may magnify the effects but may also be too challenging to implement during and after maternity leave. The study is only generalisable to lactating women with overweight and obesity, with a personal motivation of losing weight. The women had more than 3 years of education beyond high school and enjoyed the benefit of a longer paid maternal leave than in most countries.

The power analysis of the RCT was performed based on weight loss from dietary treatment, which was the primary outcome. Although we did observe several statistically significant HDL-related changes from both diet and exercise treatments as well as interactions, there is a potential lack of power and risk of type 2 errors in this secondary analysis of the trial.

Although we have performed a large number of statistical tests, many of the outcomes are correlated and aspects of the same observed changes in HDL-cholesterol. Thus, our findings are unlikely to have arisen by chance but rather from the same underlying mechanism.

The clinical relevance of the observed changes in HDL-P is unclear. Comparable numbers on changes in HDL-P subclasses are difficult to find due to differences in analytical methods, reported results and population. To our knowledge, studies relating changes in these variables to CVD are lacking.

### Conclusion

The observed effects of dietary and exercise treatments on HDL subclasses in lactating women with overweight and obesity imply a better metabolic fitness and a lower CVD risk. The dietary treatment showed more effects on HDL subclasses at 12 months than the exercise treatment, possibly associated with a 10 % weight loss. However, the clinical implications of the observed effects need further investigation.

## Supplementary material

For supplementary material accompanying this paper visit https://doi.org/10.1017/S0007114522000241. click here to view supplementary material
