# Supplementary material for: Effects of dietary and exercise treatments on HDL subclasses in lactating women with overweight and obesity: a secondary analysis of a randomised controlled trial
Source: Br J Nutr. 2022 Jan 24;128(11):2105–14. doi: 10.1017/S0007114522000241 (PMC9661371; doi:10.1017/S0007114522000241)
Supplement: Supplementary file 1 [file S0007114522000241sup001.pdf]

Supplementary table 1. Main and interaction effects of dietary and exercise treatments on lipid content in HDL subclasses in lactating women with overweight and obesity.

Lipid content at 3 months		Estimated Marginal Means (SE)				*P* main effect		*P* inter-action effect
		Diet n=31	No diet n=31	Exercise n=32	No exerc. n=30	Diet	Exercise	
**XL-HDL (mmol/l)**	TL	-0.029 (0.015)	-0.067 (0.015)	-0.013 (0.015)	-0.083 (0.015)	0.091	**0.002**	0.934
	PL	-0.013 (0.008)	-0.035 (0.008)	-0.007 (0.008)	-0.041 (0.008)	0.064	**0.004**	0.810
	C	-0.014 (0.007)	-0.030 (0.007)	-0.004 (0.007)	-0.04 (0.008)	0.145	**0.001**	0.867
	CE	-0.010 (0.005)	-0.020 (0.005)	-0.002 (0.005)	-0.028 (0.006)	0.176	**0.002**	0.822
	FC	-0.004 (0.002)	-0.009 (0.002)	-0.002 (0.002)	-0.012 (0.002)	0.099	**<0.001**	0.991
	TG	-0.002 (0.001)	-0.003 (0.001)	-0.003 (0.001)	-0.002 (0.001)	0.479	0.530	0.223
**L-HDL (mmol/l)**	TL	-0.034 (0.031)	-0.084 (0.031)	-0.058 (0.030)	-0.060 (0.031)	0.260	0.965	0.225
	PL	-0.021 (0.015)	-0.038 (0.015)	-0.033 (0.014)	-0.027 (0.015)	0.427	0.763	0.171
	C	-0.009 (0.015)	-0.043 (0.015)	-0.021 (0.015)	-0.030 (0.016)	0.133	0.672	0.341
	CE	-0.007 (0.012)	-0.033 (0.012)	-0.016 (0.012)	-0.024 (0.012)	0.135	0.673	0.319
	FC	-0.002 (0.004)	-0.010 (0.004)	-0.005 (0.003)	-0.007 (0.004)	**0.040**	0.667	0.423
	TG	-0.003 (0.001)	-0.004 (0.001)	-0.005 (0.001)	-0.002 (0.001)	0.402	0.150	0.104
**M-HDL (mmol/l)**	TL	-0.055 (0.021)	-0.024 (0.021)	-0.065 (0.020)	-0.014 (0.021)	0.299	0.087	**0.043**[a]
	PL	-0.025 (0.009)	-0.010 (0.009)	-0.028 (0.008)	-0.007 (0.009)	0.220	0.088	**0.023**[b]
	C	-0.023 0.012)	-0.012 (0.012)	-0.031 (0.012)	-0.004 (0.012)	0.515	0.131	0.110
	CE	-0.017 (0.010)	-0.009 (0.010)	-0.024 (0.010)	-0.003 (0.010)	0.569	0.144	0.132
	FC	-0.006 (0.002)	-0.003 (0.002	-0.007 (0.002)	-0.001 (0.002)	0.330	0.093	0.053
	TG	-0.007 (0.001)	-0.002 (0.001)	-0.006 (0.001)	-0.002 (0.001)	**0.006**	0.058	**0.009**[c]
**S-HDL (mmol/l)**	TL	-0.023 (0.012)	0.026 (0.012)	-0.021 (0.012)	0.024 (0.012)	**0.005**	**0.010**	0.059
	PL	-0.011 (0.007)	0.012 (0.007)	-0.012 (0.007)	0.012 (0.007)	**0.017**	**0.012**	0.013[d]
	C	-0.010 (0.007)	0.013 (0.007)	-0.007 (0.007)	0.010 (0.007)	**0.017**	0.078	0.484
	CE	-0.010 (0.006)	0.009 (0.006)	-0.006 (0.006)	0.005 (0.006)	**0.017**	0.167	0.680
	FC	<-0.001 (0.001)	0.004 (0.001)	-0.001 (0.001)	0.005 (0.002)	0.089	**0.009**	0.113
	TG	-0.002 (0.001)	0.002 (0.001)	-0.001 (0.001)	0.002 (0.001)	**0.040**	0.089	0.248

Lipid content at 12 months		Estimated Marginal Means (SE) Diet n=29	No diet n=28	Exercise n=31	No exerc. n=26	*P* main effect Diet	Exercise	P inter-action effect
**XL-HDL (mmol/l)**	TL	-0.065 (0.020)	-0.147 (0.020)	-0.077 (0.019)	-0.136 (0.021)	**0.005**	**0.040**	0.703
	PL	-0.034 (0.011)	-0.079 (0.011)	-0.042 (0.010)	-0.071 (0.011)	**0.004**	0.063	0.617
	C	-0.031 (0.009)	-0.066 (0.009)	-0.034 (0.009)	-0.063 (0.010)	**0.012**	**0.031**	0.799
	CE	-0.020 (0.007)	-0.045 (0.007)	-0.022 (0.006)	-0.043 (0.007)	**0.013**	**0.036**	0.872
	FC	-0.011 (0.003)	-0.021 (0.003)	-0.012 (0.003)	-0.020 (0.003)	**0.011**	**0.026**	0.627
	TG	<0.001 (0.001)	-0.003 (0.001)	-0.001 (0.001)	-0.002 (0.001)	**0.016**	0.376	0.794
**L-HDL (mmol/l)**	TL	-0.045 (0.032)	-0.202 (0.033)	-0.117 (0.031)	-0.130 (0.034)	**0.001**	0.789	0.899
	PL	-0.025 (0.015)	-0.095 (0.015)	-0.060 (0.014)	-0.059 (0.016)	**0.002**	0.957	0.940
	C	-0.022 (0.017)	-0.104 (0.017)	-0.057 (0.0016)	-0.069 (0.018)	**0.002**	0.646	0.850
	CE	-0.018 (0.013)	-0.079 (0.013)	-0.044 (0.012)	-0.053 (0.014)	**0.002**	0.647	0.884
	FC	-0.005 (0.004)	-0.024 (0.004)	-0.013 (0.004)	-0.016 (0.004)	**0.001**	0.644	0.743
	TG	0.002 (0.001)	-0.004 (0.001)	<0.001 (0.001)	-0.001 (0.001)	**0.005**	0.488	0.841
**M-HDL (mmol/l)**	TL	-0.059 (0.022)	-0.110 (0.022)	-0.113 (0.021)	-0.056 (0.023)	0.051	0.073	0.881
	PL	-0.024 (0.009)	-0.044 (0.009)	-0.044 (0.008)	-0.024 (0.009)	0.116	0.105	0.588
	C	-0.032 (0.013)	-0.066 (0.013)	-0.067 (0.013)	-0.031 (0.014)	0.069	0.065	0.764
	CE	-0.026 (0.015)	-0.054 (0.011)	-0.055 (0.010)	-0.026 (0.011)	0.072	0.059	0.703
	FC	-0.005 (0.003)	-0.012 (0.003)	-0.012 (0.002)	-0.006 (0.003)	0.064	0.104	0.967
	TG	-0.003 (0.001)	-0,0001 (0.002)	-0.002 (0.002)	-0.001 (0.002)	0.176	0.753	0.110
**S-HDL (mmol/l)**	TL	0.007 (0.023)	0.014 (0.016)	-0.008 (0.017)	0.030 (0.017)	0.763	0.103	0.761
	PL	-0.007 (0.008)	0.000 (0.008)	-0.017 (0.008)	0.009 (0.009)	0.552	**0.031**	0.619
	C	0.011 (0.009)	0.007 (0.009)	0.003 (0.009)	0.014 (0.010)	0.741	0.387	0.278
	CE	0.008 (0.008)	0.004 (0.008)	0.003 (0.007)	0.009 (0.008)	0.737	0.572	0.258
	FC	0.003 (0.002)	0.003 (0.002)	0.001 (0.002)	0.006 (0.002)	0.863	0.086	0.537
	TG	0.003 (0.002)	0.009 (0.002)	0.006 (0.002)	0.006 (0.002)	**0.022**	0.988	0.345

Abbreviations: SE=standard error, XL=very large, L=large, M=medium, S=small, TL = Total lipids, PL = Phospolipids, C = Cholesterol, CE= Cholesterol esters, FC = Free cholesterol, TG = Triglycerides

[a]Significant interaction effect in M-HDL-PL at 3 months. Simple main effects of diet significant within non-exercise groups (P=0.016). EMM (SE) non-diet, non-exercise= 0.014 (0.012). EMM (SE) diet, non-exercise= -0.029 (0.012). Simple main effect of exercise significant within non-diet groups (P=0.006)). EMM (SE) non-exercise, non-diet= 0.014 (0.012). EMM (SE) exercise, non-diet= -0.035 (0.012)).

[b]Significant interaction effect in M-HDL-TL at 3 months. Simple main effects of diet significant within non-exercise groups (P=0.035). EMM (SE) non-diet, non-exercise= 0.032 (0.030). EMM (SE) diet, non-exercise=-0.060 (0.030). Simple main effect of exercise significant within non-diet groups (P=0.009)). EMM (SE) non-exercise, non-diet= 0.032 (0.030). EMM (SE) exercise, non-diet= -0.080 (0.029)).

[c]Significant interaction effect in M-HDL-TG at 3 months. Simple main effects of diet significant within non-exercise groups (P<0.001). EMM (SE) non-diet, non-exercise= 0.003 (0.002). EMM (SE) diet, non-exercise=-0.007 (0.002). Simple main effect of exercise significant within non-diet groups (P=0.002)). EMM (SE) non-exercise, non-diet= 0.003 (0.002). EMM (SE) exercise, non-diet= -0.006 (0.002)).

[d]Significant interaction effect in S-HDLPL at 3 months. Simple main effects of diet significant within non-exercise groups (P<0.001). EMM (SE) non-diet, non-exercise= 0.036 (0.010). EMM (SE) diet, non-exercise=-0.011 (0.010). Simple main effect of exercise significant within non-diet groups (P<0.001). EMM (SE) non-exercise, non-diet=-0.036 (0.010). EMM (SE) exercise, non-diet=-0.012 (0.010)).
